# Central Institute of Psychiatry: A tradition in excellence

**DOI:** 10.4103/0019-5545.42405

**Published:** 2008

**Authors:** S. Haque Nizamie, Nishant Goyal, Mohammad Ziaul Haq, Sayeed Akhtar

**Affiliations:** Department of Psychiatry, Central Institute of Psychiatry, Kanke (PO), Ranchi - 834 006, Jharkhand, India

## INTRODUCTION

Growth of mental asylums in British India was a less conspicuous form of social control which reflected the colonial mindset of the prevailing societal norms.[1] The first lunatic asylum in India was established in Bombay (Mumbai) in the year 1745, followed by Calcutta (Kolkata) in 1784. Subsequently number of such asylums increased significantly and by the year 1947, there were 31 mental hospitals in India.[2] After independence mental hospitals were opened in various states. A recent survey revealed that there are 59 mental hospitals in the country.[3] Central Institute of Psychiatry (CIP), Ranchi is a premier institute for mental health in India. It started in 1918 and since then it has been a pioneer in the field of psychiatry. Till independence, this hospital was meant exclusively for the treatment of European patients. With a bed capacity of 643, it is spread over an area of 210 acres and the wards are named after eminent European psychiatrists, e.g., Kraepelin, Bleuler, Freud, Maudsley, etc. Recent facilities and establishments are named after eminent Indian psychiatrists like D. Satyanand, L. P. Verma, R. B. Davis, K. Bhaskaran, etc. It may be worth noting that unlike other mental hospitals, CIP has never been a custodial care facility. It has always been an open hospital with a comprehensive biopsychosocial approach for management of mentally ill patients. Currently, the institute functions under the administrative control of Directorate General of Health Services, Ministry of Health and Family Welfare, New Delhi, with the objectives of patient care, manpower development, and research in the field of mental health.

## DEVELOPMENT OF CIP: 1918-1947

In India, the traditional approach for the care of mentally ill patients during the last 200 years has been modeled after contemporary Britain, being custodial in nature.[4] Patients were treated at secluded places in unhygienic conditions without any facilities to fulfill their basic needs. The condition worsened in the early years of twentieth century when there was substantial increase in the number of patients during World War I. By the end of 1914, it was recognized that the lunatic asylums at Bhowanipore and Berhampore in Bengal were in bad shape, overcrowded with European patients. A dire need to establish a new asylum to accommodate these patients was felt. Ranchi was proposed as the preferred site for construction of a mental asylum probably for its location, climate, and wilderness at that time. Writing about inception of this hospital, Lt. Colonel Owen Berkeley-Hill[5] wrote:

“*To the best of my knowledge Ranchi European Asylum as it was first called was the product of a panic on the part of the government of Bengal… people of Calcutta were beginning to realize that the old Bhowanipore asylum was a disgrace to their fair city. I know as a fact that round about 1880 Indian lunatics in Bhowanipore were employed in dragging scavenger carts through the streets. Guilty conscience in Calcutta grew so numerous that at least it was decided that some thing should be done about it.*”

The ravaging condition of the lunatics caused a lot of stir in Calcutta and after much deliberation the work started at Kanke, Ranchi and by 1918 the asylum destined for Europeans and Anglo-Indians was ready to receive its guests. Ranchi European Lunatic Asylum came into existence on May 17, 1918 and all Europeans and Anglo-Indian inmates of Bhowanipore and Berhampore Lunatic Asylum were transferred here.[6] At the outset, it had a bed capacity for 174 patients and it was under the direct control of Government of Bihar. On October 27, 1919 Lt. Colonel Owen Berkeley-Hill, a psychiatrist in the British Army became the medical superintendent of the European Lunatic Asylum. With great efforts, he could manage to convince the authorities to change names of ‘*lunatic asylum* ‘ in India to ‘*mental hospital* ‘ in 1920.[7] In 1922, the institute was named as European Mental Hospital and it was put under the control of a Board of Trustees with various participating state governments represented in the Board. This year was also notable for the fact that the hospital got affiliation from the University of London for the Diploma in Psychological Medicine examination.[7] It was a unique phenomenon since postgraduate medical training was nonexistent in India in those days. In the year 1925, Ranchi also got a unique gift to the cause of mental health as Indian Mental Hospital (now called RINPAS) was created to cater Indian patients and Captain J. E. Dhunjibhoy became its first medical superintendent. This marked a major leap in mental health in this region.[6]

Berkeley-Hill remained medical superintendent of this hospital for 15 years during which this place saw some of the most notable developments. Being a hospital exclusively for Europeans, this hospital acquired all latest facilities for the management of patients within short time of their inception in the western world. Thus, it has many firsts to its credit in the development of mental health services and research in India.

The medical staff at the outset consisted of five medical officers and a compounder who catered to 79 male and 69 female patients. Initially, patients were managed using rest, morphia and organotherapy (using adrenal, liver, pituitary, gonad, and thyroid extracts) and were provided with facilities for exercise, excursions, and amusements.[6] The hospital had a musical band and patients used to go to a weekly social - an evening with dance, music, eats, and mixing of sexes. Privilege to attend socials was used to control undesirable behaviors. Hydrotherapy started in 1923 in which patients suffering from acute excitement were treated with prolonged immersion in water at an agreeable temperature in baths of special design. During the same time, the hospital started to raise interest of public in mental hygiene and prophylaxis, taking initiatives in preventive aspects of psychiatry. A “*welfare inquiry letter*” was sent to all discharged patients every 6 months to ensure their well-being [Illustration 1].[8]

**Illustration 1:**
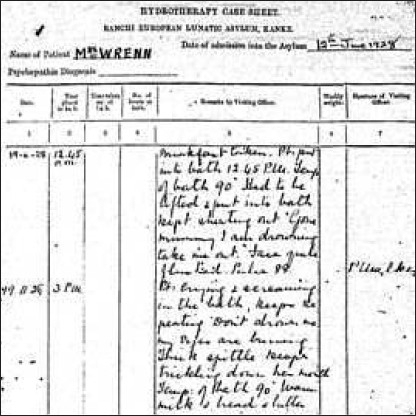
Hydrotherapy case sheet in 1928

The origins of the Psychiatric Rehabilitation in India can be traced to innovative service programs which were initiated in 1922 when Occupational Therapy Unit started at this place. It was a landmark being first such establishment in the country. In 1929, Cottages were built outside the hospital in the vicinity to keep patients with their family members for family therapy. Techniques similar to token-economy were first started in 1920 and called by the name “Habit Formation Chart” [Illustration 2].[9,10]

**Illustration 2:**
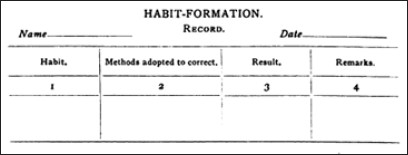
Habit Formation Chart (Taken from: Berkeley-Hill, O. 1924)

After Girindrashekhar Bose founded the Indian Psychoanalytical Association in 1922 in Calcutta, Berkeley-Hill started the Indian Association for Mental Hygiene at Ranchi.[7] He was one of the earliest practitioners of psychoanalysis in India who used this technique to help British patients to adjust to their lives after the ravages of World War I [Illustration 3].[11]

**Illustration 3:**
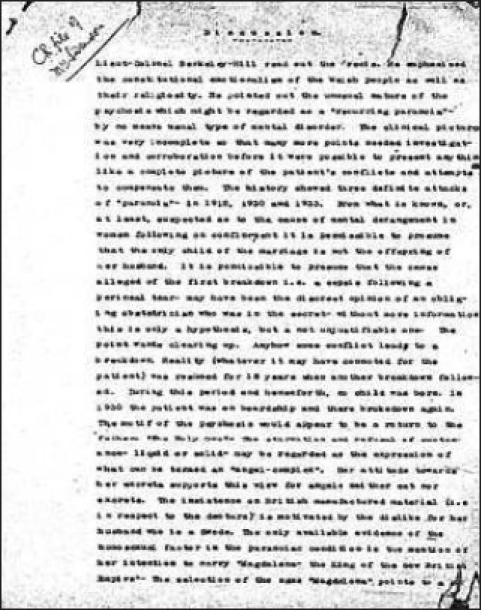
Psychoanalysis Report of a Patient by Berkeley-Hill in 1933

Development of convulsive therapy as an effective therapeutic modality started taking place in Europe in 1937 when von Meduna first described results of chemical induction of seizures to manage acute excitement. This was one of the first centers outside Europe to start Cardiazol-induced seizure treatment in 1938 and electroconvulsive therapy (ECT) in 1943 ushering a new era for treatment of severe mental disorders [Illustration 4]. ECT was given using a machine made by Wilcox and Friedman, based on AC current called the Ediswan System.[10,12] It was the most commonly used ECT system in the world at that time [Illustration 5]. Development of psychopharmacology was preceded by use of Rauwolfia extracts in the form of Santina, Serpasil, and Meralfen for psychotic conditions in late 1940s. Treatment of epilepsy and associated mental comorbidity have been an integral part of therapeutic services provided at this place. Epilepsy was regularly monitored using seizure logging charts [Illustration 6]. Merritt and Putnam discovered usefulness of phenytoin for controlling seizures in 1938[13] and the very next year it was available for treatment of epilepsy here. Medications like Primidone (Mysoline) were also used within 2 years of their introduction in the western world [Illustration 7]. The institute took initiatives in manpower development and research at the very outset. Dr. L. P. Varma, the first MD in Psychiatry (1943) in the country got degree from CIP under Patna University. A library on mental health started in 1918 with 300 books and journals which dated back from 1910.[8]

**Illustration 4:**
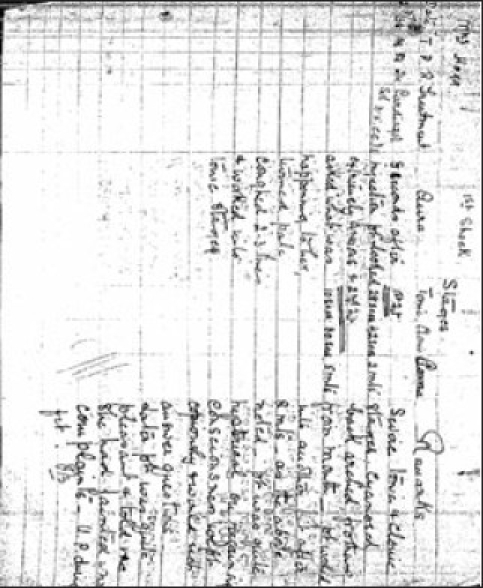
Cardiazol Therapy in 1938

**Illustration 5:**
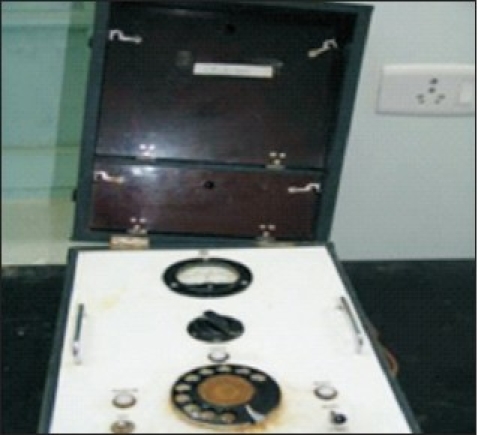
Ediswan System for ECT

**Illustration 6:**
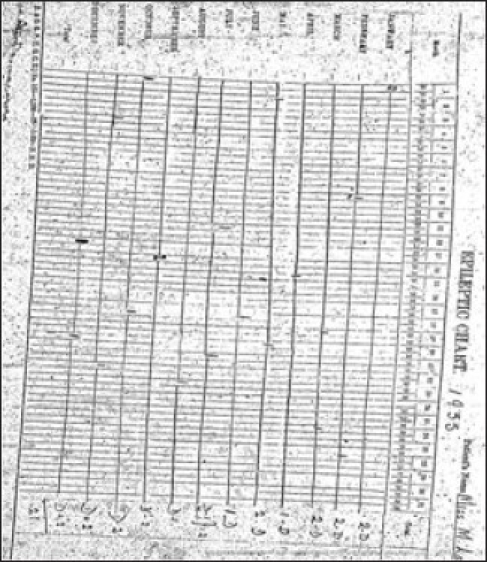
Use of Seizure Logging Charts in 1935

**Illustration 7:**
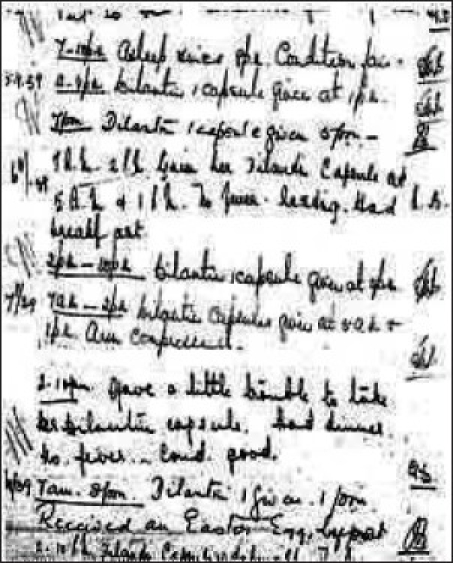
Use of Dilantin (Phenytoin) in 1939

## JOURNEY FROM MENTAL HOSPITAL TO INSTITUTE: 1947-1977

After independence, the name of European Mental Hospital was changed to Inter-Provincial Mental Hospital in 1948 and the hospital was opened for all Indians. It was subsequently renamed as Hospital for Mental Diseases in 1952 and in 1954 its administration was taken over by the Government of India.[7] Despite increasing patient load and changing social scenarios, the institute continued to follow a trend of excellence in its growth. The department of neurophysiology was established in 1948 with a 6–channel Ediswan make electroencephalography (EEG) machine and subsequently an 8–channel EEG machine was installed in 1954.[10] During the same period, a modern Radiology department with facility for cerebral angiography, pneumoencephalography, air ventriculography, myelography, etc. was established. Psychosurgery first performed by Egas Moniz, in 1935, was first used in India as a method of treatment for intractable psychosis in Bombay.[14] Psychosurgery started at Ranchi in 1947 when first Prefrontal Leucotomy was performed [Illustration 8]. Transorbital Leucotomy was first performed in India in 1949 in this institute on a patient suffering from chronic intractable psychosis 2 years after the actual procedure described by Freedman. The surgical procedures were backed up with a fully equipped neuropathology section established in 1952. Neurosurgery also started during the same period. Penfield first described electrocorticography in 1939 to lateralize seizure origin in a patient with bitemporal epilepsy.[15] This heralded the phenomenal growth of intraoperative electrical monitoring of brain all over the world. This procedure was routinely performed at this place post-1948 in patients who were planned for neurosurgery and tumor removal. Some of the tumors removed in those days are still preserved in the Department of Pathology. Psychopharmacology changed the face of psychiatric treatment with the advent of chlorpromazine in 1950s.[16] In early phase of development of psychotropic drugs, Ranchi was the first place to use chlorpromazine in India in 1953. Similarly after the introduction of lithium by Cade in 1949, this was the first center in India that used lithium in 1952 [Illustration 9].[10] The Department of Clinical Psychology started in 1949 and it happens to have the first clinical psychology laboratory in the country. Over years, the department has gained wide recognition for its high quality teaching, clinical practice, and research. The institute started a Child Guidance Clinic in 1950 followed by an independent 50-bed child and adolescent psychiatry unit in 1975. Since then child psychiatry has been an important discipline at CIP. It took initiatives in community mental health services as one of the earliest rural mental health clinic was started at Mandar near Ranchi in 1967. Industrial psychiatric unit started at Heavy Engineering Corporation (HEC) at Hatia, Ranchi in 1973. The institute also played pivotal role in establishing Indian Psychiatric Society as Major R. B. Davis was one of the founder members and the first treasurer of the society. Moore Taylor, then medical superintendent was one of the members of Bhore Committee who wrote the chapter on mental health in the report.[10] The first draft of Mental Health Act that subsequently became the Mental Health Act of India (1987) was written at Ranchi in 1949 by R. B. Davis, then Medical Superintendent, S. A. Hasib, from Indian Mental Hospital, Ranchi and J Roy, from Mental Hospital, Nagpur [Illustration 10].[7] The current postgraduate training courses started in 1962 with Diploma in Psychological Medicine (DPM) and Diploma in Medical and Social Psychology (DMSandP). Subsequently Diploma in Psychiatric Social Work (DPSW) started in 1970 followed by MD (Psychiatry) in 1971, PhD in Clinical Psychology in 1973, and Diploma in Psychiatric Nursing (DPN) in 1983.

**Illustration 8:**
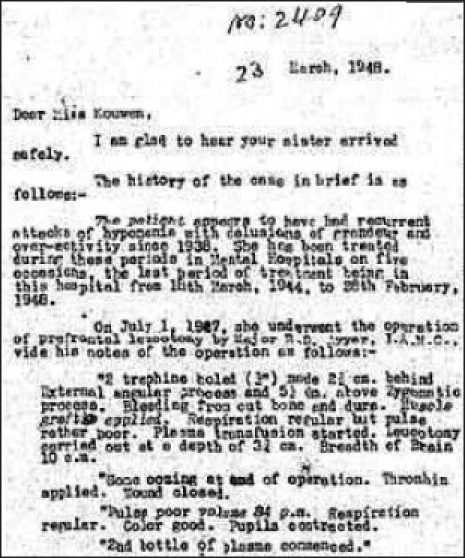
Prefrontal Leucotomy in 1947

**Illustration 9:**
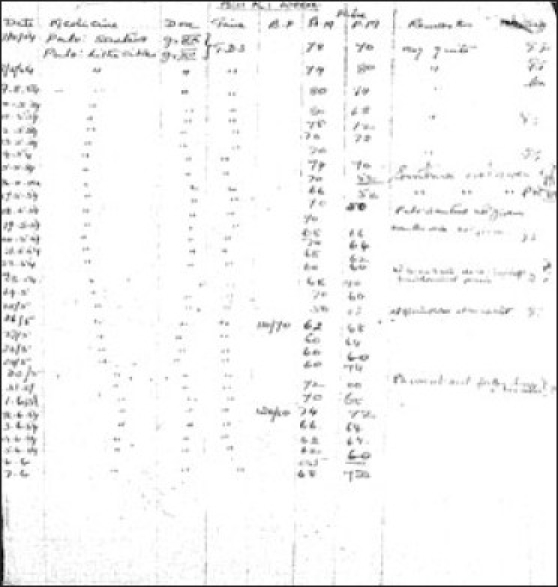
Use of Lithium Citrate in 1954

**Illustration 10:**
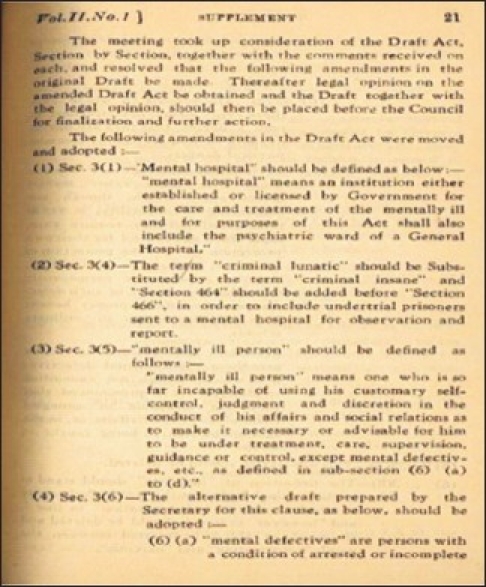
Draft of Mental Health Act at CIP in 1949

## JOURNEY TOWARD EXCELLENCE - SINCE 1977

On April 1, 1977 it was raised to the status of an Institute and was renamed as Central Institute of Psychiatry. Keeping pace with the development in mental health, the institute continued to scale new heights and various modern facilities for investigation and management of mental disorders were added.

Center for Cognitive Neurosciences at the institute has witnessed substantial growth in clinical and research applications in the field of cognitive neurosciences. In 1994, the department got 32 channels quantitative EEG and eight channels evoked potential system. In 2003, facilities for 40 channels video EEG, dense array EEG acquisition and analysis systems (64, 128, and 192 channels), event related potential (ERP) acquisition and analysis units (40 and 128 channels), Polysomnography (40 channels), and a repetitive transcranial magnetic stimulation (rTMS) were added [Illustration 11]. The center also acquired advanced softwares for sophisticated EEG analysis, e.g., Advanced Source Analysis (ASA), BESA, MATLAB, Neuroscan, etc. The Center for Neuroimaging and Radiological Sciences acquired Ultrasonography unit, Color Doppler, Transcranial Doppler, and 16 slice Spiral CT scan facility which are routinely used for clinical and research purposes. The Clinical Psychology Laboratory is well stacked with various tests for assessment of cognitive functions, personality, and other domains of human behavior, e.g., Cambridge Automated Test Battery (CANTAB), computerized version of Luria Nebraska Neuropsychological Battery (LNNB), etc. The Computer Department started in 1988 when computers were first acquired and subsequently it has been growing continuously with advances in technology. Efforts for digitalization of data and networking in the institute started in 2000. The institute started its website www.cipranchi.nic.in and a dedicated helpline service with toll-free telephone number and e-mail services. A high-speed leased line and broadband connectivity was acquired for efficient internet usage. In 2006, the institute acquired 64-Opteron cluster server for high-speed calculations and networking, enabling efficient data management and security with comprehensive interconnections at approved locations. Currently, the Medical Library at the institute provides modern facilities to postgraduate trainees and scholars with 52,000 books and bound journals, 510 print and electronic journals, high-speed Internet connectivity and photocopy facility. Library is also a member of National Medical Library's Consortia, namely, ERMED - India which provide online full text access to more than 1500 journals. The institute started providing deaddiction services from 1998 and a dedicated Center for Addiction Psychiatry started in 1999. It is a modern center with a bed capacity of 30 inpatients for treatment of alcohol and drug dependence. The institute also acts as the nodal center for implementation of National Mental Health Program (NMHP) [Illustration 12].

**Illustration 11:**
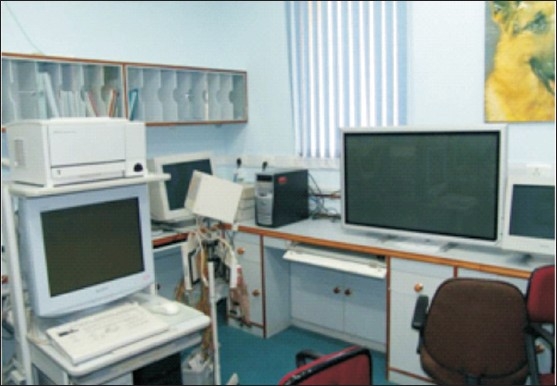
High Resolution (192 Channel) EEG System

**Illustration 12:**
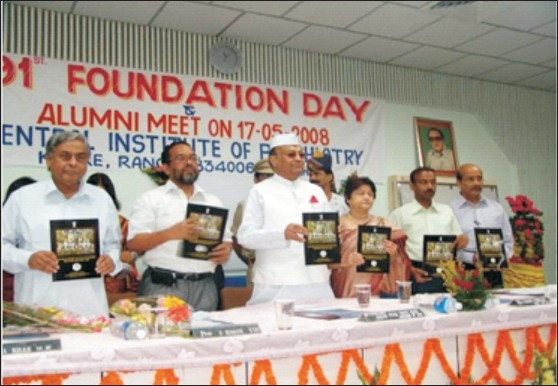
H E Governor of Jharkhand releasing CIP annual Report on the occasion of 91^st^ Foundation Day

## CONCLUSION

Ranchi has been synonymous with mental health world over. This year CIP celebrates its 91st Foundation Day. The journey has been long and distinguished and its contribution to Indian Psychiatry has set a tradition of excellence in the field of mental health. Berkeley-Hill wrote in his parting note,[5]

“*The miserable bear-garden I had taken charge of in October, 1919, had become the finest mental hospital in Asia, and a great deal finer than many mental hospitals in Europe.*”
